# Supplementary material for: MRI-Based Radiomics for Outcome Stratification in Pediatric Osteosarcoma
Source: Cancers (Basel). 2025 Aug 6;17(15):2586. doi: 10.3390/cancers17152586 (PMC12345888; doi:10.3390/cancers17152586)
Supplement: Supplementary file 1 [file cancers-17-02586-s001.zip › cancers-3755341-supplementary.pdf]

Supplementary Materials

Table S1. Selected features from classification analysis – Progressive disease

Segmentation	RF type	Selected Features	
Whole tumor	IBSI RFs	original_shape_SurfaceArea<br>original_glrlm_RunLengthNonUniformity	original_firstorder_Energy<br>original_firstorder_Skewness
	IBSI RFs + baseline clinical	Humerus<br>original_shape_SurfaceArea<br>original_glrlm_RunLengthNonUniformity<br>original_firstorder_Energy	Fibula<br>original_firstorder_Skewness<br>Skip lesion<br>Laterality
	All RFs	wavelet-HHH_original_firstorder_Maximum<br>log-sigma-3-mm-3D_original_glszm_SizeZoneNonUniformity<br>wavelet-HLL_original_firstorder_Kurtosis<br>wavelet-LLH_original_glszm_SmallAreaEmphasis<br>wavelet-LLH_original_glcm_ClusterProminence<br>wavelet-LLL_original_glszm_LargeAreaEmphasis<br>wavelet-HHH_original_glszm_SmallAreaLowGrayLevelEmphasis	wavelet-LHL_original_glszm_SizeZoneNonUniformity<br>log-sigma-3-mm-3D_original_ngtdm_Busyness<br>log-sigma-1-mm-3D_original_glszm_SizeZoneNonUniformity<br>wavelet-HHH_original_glszm_SizeZoneNonUniformity<br>wavelet-LLH_original_glszm_SmallAreaHighGrayLevelEmphasis<br>wavelet-LLH_original_firstorder_Energy
	All RFs + baseline clinical	Humerus<br>wavelet-HHH_original_firstorder_Maximum<br>log-sigma-3-mm-3D_original_glszm_SizeZoneNonUniformity<br>wavelet-HLL_original_firstorder_Kurtosis<br>log-sigma-1-mm-3D_original_glszm_SizeZoneNonUniformity<br>wavelet-LLL_original_glszm_LargeAreaEmphasis	wavelet-LHL_original_glszm_SizeZoneNonUniformity<br>log-sigma-3-mm-3D_original_ngtdm_Busyness<br>wavelet-LHH_original_glszm_SizeZoneNonUniformity<br>wavelet-LLH_original_glszm_SmallAreaEmphasis<br>wavelet-LLL_original_glszm_ZoneVariance<br>wavelet-LLH_original_glcm_ClusterProminence<br>wavelet-LHL_original_gldm_GrayLevelVariance<br>wavelet-HHH_original_glszm_SizeZoneNonUniformity
Whole tumor/ tumor sampling	Only baseline clinical	Humerus<br>Fibula<br>Skip lesion<br>Laterality<br>Raceothers<br>Femur	Chondroblastic<br>Metastasis at diagnosis<br>Hispanic<br>Age At MRI Imaging<br>Tibia<br>Osteoblastic
Tumor sampling	IBSI RFs	original_firstorder_Energy_front<br>original_glrlm_LongRunEmphasis_front<br>original_glrlm_RunVariance_bottom<br>original_glrlm_RunLengthNonUniformity_left<br>original_glrlm_RunLengthNonUniformity_middle<br>original_shape_MinorAxisLength_bottom<br>original_firstorder_Energy_top<br>original_shape_MajorAxisLength_front	original_firstorder_Minimum_top<br>original_glszm_LargeAreaEmphasis_top<br>original_glrlm_ShortRunEmphasis_left<br>original_firstorder_Energy_left<br>original_firstorder_Skewness_right<br>original_firstorder_Kurtosis_back<br>original_firstorder_Skewness_bottom
	IBSI RFs + baseline clinical	Humerus<br>original_firstorder_Energy_front<br>original_glrlm_LongRunEmphasis_front	original_glrlm_RunVariance_bottom<br>original_glrlm_RunLengthNonUniformity_left<br>original_glrlm_RunLengthNonUniformity_middle

	All RFs	log-sigma-3-mm-3D_original_firstorder_InterquartileRange_left<br>wavelet-HHH_original_firstorder_Skewness_left<br>wavelet-LHH_original_firstorder_Entropy_back<br>wavelet-HLL_original_glrlm_HighGrayLevelRunEmphasis_left<br>log-sigma-5-mm-3D_original_ngtdm_Busyness_front<br>log-sigma-5-mm-3D_original_firstorder_10Percentile_bottom<br>log-sigma-5-mm-3D_original_glszm_SmallAreaLowGrayLevelEmphasis_front	wavelet-LLH_original_firstorder_Mean_bottom<br>wavelet-LHH_original_glrlm_GrayLevelNonUniformityNormalized_front<br>wavelet-LLH_original_glcm_JointEnergy_bottom<br>log-sigma-1-mm-3D_original_glrlm_GrayLevelVariance_front<br>wavelet-LHH_original_firstorder_Entropy_left<br>wavelet-HLL_original_glszm_SmallAreaHighGrayLevelEmphasis_bottom<br>log-sigma-3-mm-3D_original_firstorder_10Percentile_back<br>wavelet-HHH_original_glcm_SumSquares_bottom
	All RFs + baseline clinical	wavelet-LLH_original_glrlm_GrayLevelVariance_top<br>humerus<br>log-sigma-5-mm-3D_original_ngtdm_Busyness_front<br>wavelet-HHH_original_firstorder_Skewness_left<br>log-sigma-3-mm-3D_original_firstorder_InterquartileRange_left<br>wavelet-LHH_original_firstorder_Entropy_back<br>wavelet-LLH_original_glcm_JointEnergy_bottom	wavelet-HLL_original_glrlm_HighGrayLevelRunEmphasis_left<br>wavelet-HHH_original_glcm_SumSquares_bottom<br>wavelet-HHL_original_glszm_SmallAreaLowGrayLevelEmphasis_top<br>wavelet-HLL_original_glszm_SmallAreaHighGrayLevelEmphasis_bottom
Bone/ soft tissue	IBSI RFs	original_glrlm_RunLengthNonUniformity_tissue	
	IBSI RFs + baseline clinical	original_glrlm_RunLengthNonUniformity_tissue	
	All RFs	log-sigma-1-mm-3D_original_glszm_SmallAreaLowGrayLevelEmphasis_bone<br>wavelet-HLL_original_glszm_SizeZoneNonUniformity_tissue<br>log-sigma-5-mm-3D_original_glszm_GrayLevelNonUniformity_tissue<br>log-sigma-5-mm-3D_original_glszm_SizeZoneNonUniformity_tissue<br>wavelet-HLH_original_firstorder_Kurtosis_tissue<br>wavelet-HHL_original_firstorder_Median_bone	wavelet-HLH_original_firstorder_Energy_tissue<br>original_glszm_ZoneVariance_tissue<br>log-sigma-5-mm-3D_original_glcm_ClusterProminence_bone<br>wavelet-HLH_original_glszm_SizeZoneNonUniformity_tissue<br>log-sigma-5-mm-3D_original_glrlm_RunLengthNonUniformity_tissue<br>log-sigma-1-mm-3D_original_firstorder_Mean_bone<br>wavelet-LHH_original_glszm_SizeZoneNonUniformity_tissue<br>log-sigma-1-mm-3D_original_firstorder_Kurtosis_tissue
	All RFs + baseline clinical	log-sigma-1-mm-3D_original_glszm_SmallAreaLowGrayLevelEmphasis_bone<br>wavelet-HLL_original_glszm_SizeZoneNonUniformity_tissue<br>log-sigma-5-mm-3D_original_glszm_GrayLevelNonUniformity_tissue<br>log-sigma-5-mm-3D_original_glszm_SizeZoneNonUniformity_tissue<br>wavelet-HLH_original_firstorder_Kurtosis_tissue<br>wavelet-HHL_original_firstorder_Median_bone<br>wavelet-HLH_original_firstorder_Energy_tissue	original_glszm_ZoneVariance_tissue<br>wavelet-HLH_original_glszm_SizeZoneNonUniformity_tissue<br>log-sigma-5-mm-3D_original_glcm_ClusterProminence_bone<br>log-sigma-5-mm-3D_original_glrlm_RunLengthNonUniformity_tissue<br>log-sigma-1-mm-3D_original_firstorder_Mean_bone<br>wavelet-LHH_original_glszm_SizeZoneNonUniformity_tissue<br>log-sigma-1-mm-3D_original_firstorder_Kurtosis_tissue<br>log-sigma-5-mm-3D_original_glszm_GrayLevelNonUniformityNormalized_tissue
	Only baseline clinical	Metastasis at diagnosis<br>Chondroblastic<br>Skip lesion<br>Raceothers<br>Humerus	Fibula<br>Telangiectatic<br>Osteoblastic<br>Laterality

Table S2. Selected features from classification analysis – Response to therapy

Segmentation	RF type	Selected Features	
Whole tumor	IBSI RFs	original_glrlm_ShortRunEmphasis<br>original_shape_Maximum3DDiameter	original_glszm_SmallAreaEmphasis
	IBSI RFs + baseline clinical	chondroblastic<br>original_glrlm_ShortRunEmphasis<br>original_glszm_SmallAreaEmphasis<br>Gender<br>original_shape_Maximum3DDiameter	original_firstorder_Energy<br>original_shape_Sphericity<br>Skip lesion<br>original_firstorder_Median
	All RFs	wavelet-LLH_original_glrlm_ShortRunEmphasis<br>wavelet-LLL_original_glszm_HighGrayLevelZoneEmphasis<br>wavelet-HLH_original_glrlm_ShortRunHighGrayLevelEmphasis<br>wavelet-LLH_original_glszm_SmallAreaLowGrayLevelEmphasis<br>wavelet-HHL_original_firstorder_Median<br>log-sigma-1-mm-3D_original_glszm_SizeZoneNonUniformityNormalized<br>wavelet-LLH_original_glszm_SizeZoneNonUniformityNormalized	log-sigma-1-mm-3D_original_glrlm_RunEntropy<br>wavelet-LHL_original_glszm_GrayLevelNonUniformityNormalized<br>wavelet-HHH_original_glrlm_LongRunLowGrayLevelEmphasis<br>log-sigma-5-mm-3D_original_glrlm_ShortRunEmphasis<br>wavelet-HLH_original_firstorder_Kurtosis<br>wavelet-HHL_original_glszm_ZoneEntropy<br>log-sigma-5-mm-3D_original_glrlm_ShortRunHighGrayLevelEmphasis
	All RFs + baseline clinical	wavelet-LLH_original_glrlm_ShortRunEmphasis<br>wavelet-LLL_original_glszm_HighGrayLevelZoneEmphasis<br>wavelet-HLH_original_glrlm_ShortRunHighGrayLevelEmphasis<br>wavelet-LLH_original_glszm_SmallAreaLowGrayLevelEmphasis<br>wavelet-HHL_original_firstorder_Median<br>log-sigma-1-mm-3D_original_glszm_SizeZoneNonUniformityNormalized<br>wavelet-LLH_original_glszm_SizeZoneNonUniformityNormalized	log-sigma-1-mm-3D_original_glrlm_RunEntropy<br>wavelet-LHL_original_glszm_GrayLevelNonUniformityNormalized<br>wavelet-HHH_original_glrlm_LongRunLowGrayLevelEmphasis<br>log-sigma-5-mm-3D_original_glrlm_ShortRunEmphasis<br>wavelet-HLH_original_firstorder_Kurtosis<br>wavelet-HHL_original_glszm_ZoneEntropy<br>log-sigma-5-mm-3D_original_glrlm_ShortRunHighGrayLevelEmphasis
Whole tumor/ tumor sampling	Only baseline clinical	Chondroblastic<br>Gender<br>Skip lesion<br>Raceothers<br>Humerus<br>Fibula<br>Racewhite<br>Raceblack	Metastasis at diagnosis<br>Telangiectatic<br>Osteoblastic<br>Tibia<br>Age At MRI Imaging<br>Hispanic<br>Femur
Tumor sampling	IBSI RFs	original_firstorder_Range_top<br>original_shape_MajorAxisLength_left<br>original_shape_Sphericity_right	original_shape_Flatness_left<br>original_gldm_DependenceEntropy_left
	IBSI RFs + baseline clinical	original_firstorder_Range_top<br>Chondroblastic<br>original_shape_Sphericity_right	Gender<br>original_shape_MajorAxisLength_left
	All RFs	wavelet-HLH_original_glszm_GrayLevelNonUniformity_left<br>wavelet-LLH_original_glszm_SmallAreaLowGrayLevelEmphasis_right<br>wavelet-HHH_original_glcm_Imc1_back<br>log-sigma-3-mm-3D_original_gldm_DependenceEntropy_right<br>wavelet-LHH_original_gldm_DependenceEntropy_right	wavelet-LHL_original_firstorder_Entropy_back<br>wavelet-HHL_original_glrlm_GrayLevelNonUniformityNormalized_left<br>wavelet-LHH_original_glszm_SizeZoneNonUniformityNormalized_left<br>wavelet-

		wavelet-HLL_original_glszm_GrayLevelNonUniformityNormalized_bottom	HLH_original_glszm_SizeZoneNonUniformityNormalized_right<br>wavelet-LHH_original_glszm_SizeZoneNonUniformity_middle
	All RFs + baseline clinical	wavelet-HLH_original_glszm_GrayLevelNonUniformity_left<br>wavelet-LLH_original_glszm_SmallAreaLowGrayLevelEmphasis_right<br>wavelet-HHH_original_glcm_Imc1_back<br>wavelet-LHH_original_gldm_DependenceEntropy_right<br>log-sigma-3-mm-3D_original_gldm_DependenceEntropy_right<br>wavelet-HLL_original_glszm_GrayLevelNonUniformityNormalized_bottom	wavelet-LHL_original_firstorder_Entropy_back<br>wavelet-HHL_original_glrlm_GrayLevelNonUniformityNormalized_left<br>wavelet-HLH_original_glszm_SizeZoneNonUniformityNormalized_right<br>wavelet-LHH_original_glszm_SizeZoneNonUniformityNormalized_left<br>wavelet-LHH_original_glszm_SizeZoneNonUniformity_middle
Bone/ soft tissue	IBSI RFs	original_firstorder_RobustMeanAbsoluteDeviation_tissue<br>original_glrlm_ShortRunEmphasis_bone<br>original_glrlm_LongRunEmphasis_bone<br>original_shape_Flatness_bone<br>original_shape_MajorAxisLength_tissue	original_glszm_SmallAreaEmphasis_tissue<br>original_glrlm_RunLengthNonUniformity_bone<br>original_glszm_ZoneVariance_tissue<br>original_glszm_LargeAreaEmphasis_tissue<br>original_firstorder_Energy_tissue
	IBSI RFs + baseline clinical	Osteoblastic<br>Chondroblastic<br>original_firstorder_RobustMeanAbsoluteDeviation_tissue<br>original_glrlm_ShortRunEmphasis_bone<br>original_glrlm_LongRunEmphasis_bone<br>original_glszm_SmallAreaEmphasis_tissue	original_shape_Flatness_bone<br>original_shape_MajorAxisLength_tissue<br>original_glszm_ZoneVariance_tissue<br>original_glrlm_RunLengthNonUniformity_bone<br>Gender<br>Hispanic
	All RFs	wavelet-LHL_original_glszm_ZonePercentage_bone<br>wavelet-LHL_original_firstorder_Kurtosis_bone	wavelet-HHL_original_glrlm_GrayLevelNonUniformityNormalized_bone<br>log-sigma-3-mm-3D_original_glszm_ZonePercentage_bone
	All RFs + baseline clinical	wavelet-LHL_original_glszm_ZonePercentage_bone<br>wavelet-LHL_original_firstorder_Kurtosis_bone	wavelet-HHL_original_glrlm_GrayLevelNonUniformityNormalized_bone
	Only baseline clinical	Osteoblastic<br>Chondroblastic<br>Hispanic<br>Gender<br>Skip lesion	Humerus<br>Fibula<br>Telangiectatic<br>Raceothers<br>Metastasis at diagnosis<br>Laterality

Table S3. Selected features from classification analysis – Relapse off therapy

Segmentation	RF type	Features	
Whole tumor	IBSI RFs	original_glszm_LargeAreaEmphasis<br>original_glrlm_RunLengthNonUniformity	original_firstorder_Energy<br>original_firstorder_Kurtosis
	IBSI RFs + baseline clinical	Metastasis at diagnosis<br>Skip Lesion	original_glszm_LargeAreaEmphasis<br>Humerus<br>Chondroblastic
	IBSI RFs + baseline clinical + prior outcomes	Metastasis at diagnosis<br>Skip Lesion	original_glszm_LargeAreaEmphasis
	All RFs	log-sigma-5-mm-3D_original_glszm_LargeAreaLowGrayLevelEmphasis<br>wavelet-LLL_original_glszm_SizeZoneNonUniformity<br>wavelet-LLL_original_glszm_ZoneVariance<br>wavelet-HLH_original_firstorder_Energy<br>wavelet-LLL_original_glszm_GrayLevelNonUniformity	wavelet-LLH_original_glszm_ZoneEntropy<br>wavelet-LLH_original_firstorder_Kurtosis<br>wavelet-HLL_original_firstorder_Mean<br>wavelet-HLL_original_glszm_SmallAreaHighGrayLevelEmphasis<br>wavelet-HHH_original_firstorder_Kurtosis<br>wavelet-HLL_original_firstorder_Maximum
	All RFs + baseline clinical	metastasis<br>wavelet-LLL_original_glszm_SizeZoneNonUniformity<br>log-sigma-5-mm-3D_original_glszm_LargeAreaLowGrayLevelEmphasis<br>Skip lesion<br>wavelet-LLL_original_glszm_ZoneVariance	wavelet-HLH_original_firstorder_Energy<br>wavelet-LLL_original_glszm_GrayLevelNonUniformity<br>wavelet-LLH_original_glszm_ZoneEntropy<br>wavelet-HLL_original_firstorder_Maximum<br>wavelet-LLH_original_firstorder_Kurtosis
	All RFs + baseline clinical + prior outcomes	Metastasis at diagnosis<br>wavelet-LLL_original_glszm_SizeZoneNonUniformity<br>log-sigma-5-mm-3D_original_glszm_LargeAreaLowGrayLevelEmphasis	Skip lesion<br>wavelet-LLL_original_glszm_ZoneVariance
Whole tumor/ tumor sampling	Only baseline clinical	Metastasis at diagnosis<br>Skip Lesion<br>Humerus<br>Gender<br>Chondroblastic	Age At MRI Imaging<br>Osteoblastic<br>Hispanic<br>Laterality
	Only baseline clinical + prior outcomes	Metastasis at diagnosis<br>Skip Lesion<br>Humerus<br>Chondroblastic<br>Gender	Age At MRI Imaging<br>Osteoblastic<br>Corrected % Necrosis
tumor sampling	IBSI RFs	original_firstorder_Kurtosis_top<br>original_firstorder_Energy_top<br>original_glszm_LargeAreaEmphasis_middle<br>original_glrlm_ShortRunEmphasis_back<br>original_glrlm_RunLengthNonUniformity_front	original_firstorder_Range_front<br>original_firstorder_Skewness_top<br>original_glszm_SmallAreaEmphasis_bottom<br>original_gldm_DependenceEntropy_front

			original_firstorder_Energy_front<br>original_firstorder_Mean_top
	IBSI RFs + baseline clinical	Metastasis at diagnosis<br>original_firstorder_Kurtosis_top<br>Skip lesion<br>original_firstorder_Energy_top<br>original_firstorder_Skewness_top<br>original_glszm_LargeAreaEmphasis_middle<br>original_firstorder_Mean_top	Chondroblastic<br>original_firstorder_Range_front<br>Humerus<br>original_firstorder_Energy_front<br>original_glrlm_ShortRunEmphasis_back<br>original_glrlm_RunLengthNonUniformity_front
	IBSI RFs + baseline clinical + prior outcomes	Metastasis at diagnosis<br>original_firstorder_Kurtosis_top<br>Skip lesion<br>original_firstorder_Energy_top<br>original_firstorder_Skewness_top<br>original_glszm_LargeAreaEmphasis_middle	Chondroblastic<br>original_firstorder_Mean_top<br>original_firstorder_Range_front<br>original_firstorder_Energy_front<br>Humerus<br>original_glrlm_ShortRunEmphasis_back<br>original_glrlm_RunLengthNonUniformity_front
	All RFs	log-sigma-1-mm-3D_original_firstorder_Skewness_right<br>wavelet-HLL_original_firstorder_Entropy_left<br>wavelet-LLH_original_gldm_DependenceVariance_front<br>wavelet-HHL_original_glszm_ZoneVariance_top<br>wavelet-HLL_original_glszm_SmallAreaLowGrayLevelEmphasis_front	log-sigma-5-mm-3D_original_glcm_Imc2_front<br>log-sigma-1-mm-3D_original_firstorder_Skewness_front<br>wavelet-HLL_original_glcm_Imc2_left<br>wavelet-HHH_original_glszm_SmallAreaLowGrayLevelEmphasis_bottom<br>log-sigma-1-mm-3D_original_glszm_GrayLevelVariance_front<br>original_glrlm_RunLengthNonUniformityNormalized_right<br>original_firstorder_Kurtosis_top<br>wavelet-LLH_original_firstorder_TotalEnergy_top<br>log-sigma-3-mm-3D_original_glszm_SmallAreaLowGrayLevelEmphasis_middle<br>wavelet-LLH_original_glrlm_GrayLevelVariance_left
	All RFs + baseline clinical	metastasis<br>log-sigma-1-mm-3D_original_firstorder_Skewness_right<br>wavelet-HLL_original_firstorder_Entropy_left<br>log-sigma-1-mm-3D_original_firstorder_Skewness_front<br>original_firstorder_Kurtosis_top	wavelet-HHL_original_glszm_ZoneVariance_top<br>wavelet-LLH_original_gldm_DependenceVariance_front<br>wavelet-HLL_original_glszm_SmallAreaLowGrayLevelEmphasis_front<br>log-sigma-5-mm-3D_original_glcm_Imc2_front<br>Skip lesion
	All RFs + baseline clinical + prior outcomes	metastasis<br>log-sigma-1-mm-3D_original_firstorder_Skewness_right<br>wavelet-HLL_original_firstorder_Entropy_left<br>log-sigma-1-mm-3D_original_firstorder_Skewness_front<br>original_firstorder_Kurtosis_top	wavelet-HHL_original_glszm_ZoneVariance_top<br>wavelet-LLH_original_gldm_DependenceVariance_front<br>wavelet-HLL_original_glszm_SmallAreaLowGrayLevelEmphasis_front<br>log-sigma-5-mm-3D_original_glcm_Imc2_front<br>Skip lesion
Bone/ soft tissue	IBSI RFs	original_glszm_ZoneVariance_tissue<br>original_firstorder_Energy_bone	original_gldm_DependenceEntropy_bone
	IBSI RFs + baseline clinical	Skip lesion	original_glszm_ZoneVariance_tissue<br>original_firstorder_Energy_bone
	IBSI RFs + baseline clinical + prior outcomes	Skip lesion	original_glszm_ZoneVariance_tissue<br>original_firstorder_Energy_bone

	All RFs	original_firstorder_InterquartileRange_tissue<br>original_glrlm_ShortRunEmphasis_tissue<br>wavelet-LLH_original_glszm_SmallAreaLowGrayLevelEmphasis_bone<br>wavelet-LHL_original_firstorder_TotalEnergy_bone<br>wavelet-HHL_original_glszm_SizeZoneNonUniformityNormalized_bone<br>log-sigma-1-mm-3D_original_glszm_SizeZoneNonUniformityNormalized_bone<br>log-sigma-5-mm-3D_original_glszm_SizeZoneNonUniformity_bone<br>log-sigma-3-mm-3D_original_glszm_GrayLevelVariance_bone	wavelet-HLH_original_glcm_SumEntropy_tissue<br>original_glszm_ZoneVariance_tissue<br>wavelet-HLL_original_glrlm_LowGrayLevelRunEmphasis_bone<br>wavelet-LLH_original_firstorder_Skewness_tissue<br>log-sigma-1-mm-3D_original_glszm_ZoneEntropy_bone<br>wavelet-LHL_original_glszm_SmallAreaHighGrayLevelEmphasis_bone<br>wavelet-HLL_original_firstorder_Skewness_bone
	All RFs + baseline clinical	Skip lesion<br>original_firstorder_InterquartileRange_tissue<br>wavelet-LLH_original_glszm_SmallAreaLowGrayLevelEmphasis_bone<br>original_glrlm_ShortRunEmphasis_tissue<br>log-sigma-1-mm-3D_original_glszm_SizeZoneNonUniformityNormalized_bone	log-sigma-3-mm-3D_original_glszm_GrayLevelVariance_bone<br>wavelet-HHL_original_glszm_SizeZoneNonUniformityNormalized_bone<br>wavelet-HLH_original_glcm_SumEntropy_tissue<br>wavelet-LHL_original_firstorder_TotalEnergy_bone<br>wavelet-LLH_original_firstorder_Skewness_tissue
	All RFs + baseline clinical + prior outcomes	Skip lesion<br>original_firstorder_InterquartileRange_tissue<br>wavelet-LLH_original_glszm_SmallAreaLowGrayLevelEmphasis_bone<br>original_glrlm_ShortRunEmphasis_tissue<br>log-sigma-1-mm-3D_original_glszm_SizeZoneNonUniformityNormalized_bone	wavelet-HHL_original_glszm_SizeZoneNonUniformityNormalized_bone<br>log-sigma-3-mm-3D_original_glszm_GrayLevelVariance_bone<br>wavelet-HLH_original_glcm_SumEntropy_tissue<br>wavelet-LHL_original_firstorder_TotalEnergy_bone<br>wavelet-LLH_original_firstorder_Skewness_tissue
	Only baseline clinical	Skip Lesion<br>Age At MRI Imaging<br>Metastasis<br>Humerus<br>Fibula<br>Hispanic	Raceothers<br>Telangiectatic<br>Osteoblastic<br>Laterality<br>Racewhite
	Only baseline clinical + prior outcomes	Skip lesion<br>Age at MRI imaging	

Table S4. Selected features from classification analysis – OS related mortality

Segmentation	RF type	Features	
Whole tumor	IBSI RFs	original_glszm_LargeAreaEmphasis<br>original_glrlm_RunLengthNonUniformity<br>original_firstorder_Energy<br>original_firstorder_Skewness<br>original_shape_MinorAxisLength	original_firstorder_Minimum<br>original_firstorder_Kurtosis<br>original_shape_Sphericity<br>original_glrlm_ShortRunEmphasis<br>original_firstorder_Range
	IBSI RFs + baseline clinical	Chondroblastic<br>Metastasis at diagnosis<br>Skip lesion<br>original_glszm_LargeAreaEmphasis	original_glrlm_RunLengthNonUniformity<br>Fibula<br>original_firstorder_Energy<br>Laterality
	IBSI RFs + baseline clinical + prior outcomes	Progressive disease<br>Relapse Off Therapy<br>Corrected % Necrosis	
	All RFs	wavelet-LLL_original_glszm_ZoneVariance<br>wavelet-LHL_original_glszm_ZoneEntropy<br>wavelet-LLL_original_glszm_LargeAreaHighGrayLevelEmphasis<br>wavelet-LHH_original_gldm_DependenceEntropy<br>wavelet-LHL_original_glszm_GrayLevelNonUniformity<br>wavelet-HHH_original_glszm_SmallAreaEmphasis<br>log-sigma-5-mm-3D_original_firstorder_Energy	wavelet-HHH_original_firstorder_Maximum<br>wavelet-LHL_original_gldm_GrayLevelVariance<br>log-sigma-1-mm-3D_original_glszm_SizeZoneNonUniformity<br>wavelet-LHH_original_glszm_ZoneEntropy<br>wavelet-HLH_original_glszm_SizeZoneNonUniformity<br>wavelet-HLL_original_firstorder_Kurtosis<br>wavelet-LLH_original_glrlm_RunEntropy<br>wavelet-LHL_original_firstorder_Median
	All RFs + baseline clinical	Chondroblastic<br>Metastasis at diagnosis<br>wavelet-LLL_original_glszm_ZoneVariance	wavelet-LLL_original_glszm_LargeAreaHighGrayLevelEmphasis<br>Skip lesion<br>wavelet-LHL_original_glszm_ZoneEntropy
	All RFs + baseline clinical + prior outcomes	Progressive disease<br>Relapse Off Therapy<br>Corrected % Necrosis	wavelet-LHL_original_glszm_ZoneEntropy<br>wavelet-LHL_original_glszm_SizeZoneNonUniformityNormalized<br>wavelet-HLH_original_glcm_Autocorrelation
whole tumor/ tumor sampling	Only baseline clinical	Chondroblastic<br>Metastasis at diagnosis<br>Skip lesion<br>Fibula<br>Laterality<br>Humerus<br>Raceblack<br>Raceothers	Racewhite<br>Femur<br>Age At MRI Imaging<br>Tibia<br>Osteoblastic<br>Gender<br>Telangiectatic

	Only baseline clinical + prior outcomes	Progressive disease<br>Relapse Off Therapy<br>Corrected % Necrosis	
tumor sampling	IBSI RFs	original_glszm_LargeAreaEmphasis_front<br>original_firstorder_Energy_front<br>original_firstorder_Minimum_top	original_glszm_SmallAreaEmphasis_bottom<br>original_glrlm_RunVariance_bottom
	IBSI RFs + baseline clinical	Chondroblastic<br>Metastasis at diagnosis<br>Skip lesion	original_firstorder_Energy_front<br>original_glszm_LargeAreaEmphasis_front<br>original_glrlm_RunLengthNonUniformity_front<br>Laterality
	IBSI RFs + baseline clinical + prior outcomes	Progressive disease<br>Relapse Off Therapy<br>Corrected % Necrosis	
	All RFs	wavelet-LHL_original_firstorder_Minimum_front<br>wavelet-LHL_original_glrlm_LowGrayLevelRunEmphasis_front<br>log-sigma-1-mm-3D_original_glszm_SmallAreaLowGrayLevelEmphasis_back<br>wavelet-LHL_original_firstorder_Kurtosis_top<br>wavelet-HLH_original_glszm_ZoneEntropy_front<br>wavelet-HLL_original_gldm_DependenceVariance_bottom	wavelet-HLH_original_gldm_DependenceNonUniformityNormalized_left<br>wavelet-HLL_original_glszm_SmallAreaLowGrayLevelEmphasis_middle<br>log-sigma-1-mm-3D_original_ngtdm_Contrast_back<br>wavelet-HLL_original_glrlm_GrayLevelNonUniformityNormalized_front<br>wavelet-HHH_original_glcm_SumSquares_front<br>wavelet-HLH_original_glszm_SizeZoneNonUniformity_front
	All RFs + baseline clinical	wavelet-LHL_original_firstorder_Minimum_front<br>metastasis<br>wavelet-LHL_original_glrlm_LowGrayLevelRunEmphasis_front	Chondroblastic<br>log-sigma-1-mm-3D_original_glszm_SmallAreaLowGrayLevelEmphasis_back
	All RFs + baseline clinical + prior outcomes	Progressive disease<br>Relapse Off Therapy<br>wavelet-LHH_original_glszm_SizeZoneNonUniformityNormalized_right<br>log-sigma-1-mm-3D_original_glszm_SmallAreaLowGrayLevelEmphasis_back	wavelet-HLL_original_glrlm_GrayLevelNonUniformityNormalized_front<br>wavelet-HLH_original_glcm_Imc2_middle<br>wavelet-LHL_original_gldm_DependenceEntropy_middle<br>wavelet-HLH_original_gldm_DependenceNonUniformityNormalized_left
Bone/ soft tissue	IBSI RFs	original_glrlm_RunLengthNonUniformity_tissue<br>original_shape_MajorAxisLength_tissue<br>original_glszm_ZoneVariance_tissue<br>original_firstorder_Kurtosis_bone	original_firstorder_Skewness_bone<br>original_shape_MinorAxisLength_bone<br>original_shape_Sphericity_tissue
	IBSI RFs + baseline clinical	original_glrlm_RunLengthNonUniformity_tissue<br>original_shape_MajorAxisLength_tissue<br>original_glszm_ZoneVariance_tissue<br>original_firstorder_Kurtosis_bone	original_firstorder_Skewness_bone<br>original_shape_MinorAxisLength_bone<br>metastasis<br>original_shape_Sphericity_tissue

	IBSI RFs + baseline clinical + prior outcomes	Progressive disease<br>original_glrlm_RunLengthNonUniformity_tissue<br>Gender<br>original_shape_MinorAxisLength_bone	original_firstorder_InterquartileRange_bone<br>original_firstorder_Median_tissue<br>original_firstorder_RootMeanSquared_bone<br>original_shape_Sphericity_tissue
	All RFs	log-sigma-1-mm-3D_original_glrlm_RunEntropy_bone<br>wavelet-HLL_original_glszm_SizeZoneNonUniformity_tissue<br>log-sigma-5-mm-3D_original_glszm_SizeZoneNonUniformity_tissue	wavelet-LLH_original_glszm_ZoneEntropy_bone<br>original_glrlm_RunLengthNonUniformity_tissue<br>log-sigma-5-mm-3D_original_glcm_ClusterShade_tissue
	All RFs + baseline clinical	log-sigma-1-mm-3D_original_glrlm_RunEntropy_bone<br>log-sigma-5-mm-3D_original_glszm_SizeZoneNonUniformity_tissue<br>wavelet-HLL_original_glszm_SizeZoneNonUniformity_tissue	wavelet-LLH_original_glszm_ZoneEntropy_bone<br>original_glrlm_RunLengthNonUniformity_tissue<br>log-sigma-5-mm-3D_original_glcm_ClusterShade_tissue
	All RFs + baseline clinical + prior outcomes	Progressive disease<br>log-sigma-3-mm-3D_original_firstorder_RobustMeanAbsoluteDeviation_tissue	log-sigma-1-mm-3D_original_glrlm_RunEntropy_bone<br>log-sigma-5-mm-3D_original_glcm_Imc1_bone
	Only baseline clinical	Metastasis at diagnosis<br>Skip Lesion<br>Chondroblastic<br>Humerus<br>Fibula<br>Osteoblastic<br>Laterality<br>Raceothers	Telangiectatic<br>Hispanic<br>Femur<br>Tibia<br>Age At MRI Imaging<br>Raceblack<br>Gender
	Only baseline clinical + prior outcomes	Progressive disease<br>Gender	Corrected % necrosis<br>Laterality
